# Combination Immunosuppressive Therapy in Primary Autoimmune Inner Ear Disease in Pregnancy

**DOI:** 10.1155/2022/9210780

**Published:** 2022-03-18

**Authors:** Saikrishna Ananthapadmanabhan, Joe Jabbour, David Brown, Vanaja Sivapathasingam

**Affiliations:** ^1^Department of Otolaryngology, Nepean Hospital, Kingswood, NSW 2747, Australia; ^2^Department of Immunopathology, NSW Health Pathology-ICMPR, Westmead Hospital, Westmead, NSW, Australia; ^3^Sydney Medical School, University of Sydney, Sydney, NSW, Australia; ^4^Centre for Immunology and Allergy Research, The Westmead Institute for Medical Research, Westmead, Australia

## Abstract

**Objective:**

Autoimmune inner ear disease (AIED) is a rare disorder characterized by rapidly progressive, sensorineural hearing loss that demonstrates good responsiveness to corticosteroid and immunosuppressive therapy. The pathophysiology is likely driven by chronic trafficking of immune cells into the inner ear, targeting inner ear proteins to coordinate inflammation. Suppression or modulation of the immune response can minimize cochleitis allowing for potential recovery of hearing. It is an otologic emergency requiring a multidisciplinary approach to management to commence immunosuppressive therapy. This can be achieved using steroids, immunomodulators, plasmapheresis, intravenous immunoglobulin, or biologic agents. Treatment decisions are further complicated in pregnancy and require supervision by an obstetrician and maternal-fetal medicine (MFM) specialist. Concerns include safe dosing of steroids and potential for transplacental migration of immune complexes. We provide the first comprehensive literature review on AIED and its implications in pregnancy. We frame our discussion in the context of the second reported case of primary AIED in pregnancy and the first to show excellent response to immunosuppressive therapy.

**Methods:**

We reviewed the presented case and literature on AIED.

**Results:**

A 27-year-old, pregnant, HSP-70 positive woman was diagnosed with AIED and had excellent recovery of hearing and balance following a combination of steroid treatment, augmented by oral immunomodulators, plasmapheresis, and IVIG.

**Conclusion:**

AIED is a diagnostic challenge, and treatment considerations are complex when encountered in pregnancy. Management requires multidisciplinary involvement between otolaryngologists, immunologists, and obstetricians to balance maternal and fetal health outcomes.

## 1. Introduction

Autoimmune inner ear disease (AIED) is an exceptionally rare occurrence during pregnancy. It presents challenging diagnostic and management paradigms. AIED is defined as rapidly progressive, typically bilateral, hearing loss that demonstrates responsiveness to corticosteroid and immunosuppressive therapy as demonstrated by McCabe in 1979 [1, 2]. It was first described by Lehnhardt and Cogan in 1958 [3, 4]. Clinically, symptoms develop over weeks to months, which is too rapid for presbycusis and too gradual for sudden SNHL.

AIED may be primary, in which the disease process is confined to the inner ear, or secondary, accounting for 30% of cases, in which cochleovestibular damage occurs in the context of a systemic autoimmune process with circulating immune complexes [5]. Inner ear involvement secondary to multisystem inflammation has been seen in multiple conditions including Cogan's syndrome, rheumatologic conditions, and granulomatous disease [5]. The underlying immunological mechanisms are not well elucidated in the literature [6]. Autoimmune conditions may manifest during pregnancy, triggered by hormonal and physiological changes [7]. AIED should be recognized as an otologic emergency with urgent multidisciplinary management coordinated by otolaryngologists and immunologists in initiating immunosuppressive treatment that improves chance of hearing and balance recovery. Disease manifesting in pregnancy requires specialty input from obstetricians and MFM specialists [8] as treatment considerations must balance maternal and fetal health.

## 2. Case Presentation

A 27-year-old pregnant female, in the 19th week of gestation, presented to the outpatient ENT clinic with a 1-week history of bilateral SNHL, vertigo, and tinnitus. She had presented to the emergency department the previous week with flu-like symptoms, headache, photophobia, and neck stiffness, when she was discharged with a presumptive diagnosis of migraines. She was otherwise well with no medical history or regular medications. Her obstetric history was G9P1, including 6 miscarriages, 2 terminations, and 1 term pregnancy. There was no personal or family history of autoimmune disease, vasculitis, thrombophilia, or genetic conditions.

Otoscopy revealed patent external auditory canals, intact and noninflamed tympanic membranes, and no evidence of middle ear pathology. She was unsteady in her gait. There was horizontal nystagmus on left lateral gaze, and head impulse testing was positive to the left. Free field voice testing indicated bilateral hearing impairment. Tuning fork assessment was equivocal for Weber's test and Rinne's positive bilaterally. The remainder of the neurological examination was unremarkable.

**Figure 1:**
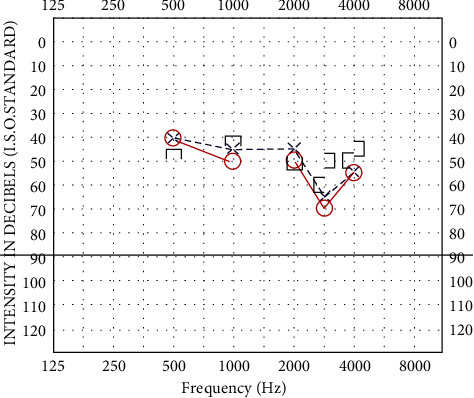
Audiogram pretreatment: bilateral mild sloping to moderate-severe sensorineural hearing loss.

Baseline pure tone audiogram showed bilateral moderate sensorineural hearing loss with flat morphology, affecting low and high frequencies (Figure 1). Speech discrimination was good with an appropriate level of amplification.

The provisional diagnosis of bilateral acute cochleovestibular inflammation was made, and urgent multidisciplinary consultations were sought from obstetrics, MFM, neurology and immunology.

The lumbar puncture showed CSF-restricted oligoclonal bands and pleocytosis with mononuclear cells (Table 1). Autoimmune encephalitis was considered based on Graus criteria [9].

An urgent MRI brain and spine was arranged which showed normal cerebellopontine angles, vestibulocochlear nerves, labyrinthine signal intensity, and architecture, with the absence of demyelination features. Serial imaging taken 2 weeks apart excluded interval cochlear fluid signal changes. Blood tests showed elevated acute phase reactants and excluded viral etiology (Tables 2–4). Autoimmune screen returned speckled antinuclear antibodies in 1 : 640 and positive HSP-70 antibodies (Table 5). The diagnosis of primary AIED was established.

**Figure 2:**
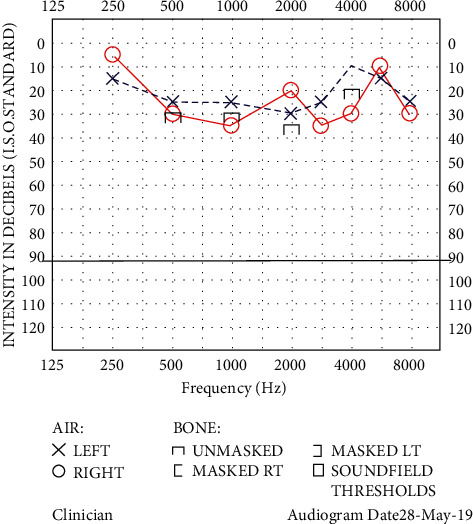
Audiogram posttreatment: mild sensorineural hearing loss (right greater than the left).

Systemic steroid treatment was commenced immediately following neurology, immunology, and MFM consultations. The patient received intravenous methylprednisolone pulsing with 1 g daily for three days. She received intravenous acyclovir 900 mg three times daily for three days as prophylactic HSV coverage on advice from neurologists. Obstetric and MFM advised on safety of medications in pregnancy. She received one dose of intratympanic dexamethasone, by which state there was marked improvement in her balance and hearing thresholds (Figure 2). Ophthalmology review excluded ocular inflammation. She was discharged on 100 mg daily azathioprine and 50 mg daily prednisolone, which was slowly weaned under immunology guidance. She remained under the combined supervision of the MFM, immunology, and ENT disciplines throughout her pregnancy. Her hearing and balance remained stable on maintenance therapy. Due to concerns of poor fetal growth, she received five sessions of plasmapheresis followed by monthly IVIG therapy.

**Figure 3:**
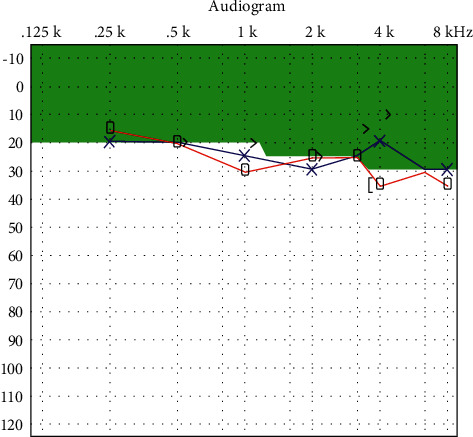
Audiogram two years posttreatment: hearing thresholds returned to baseline with mild sensorineural hearing loss at higher frequencies.

A healthy baby was born at term. The patient's newborn initially failed the newborn hearing test but passed the following week. There are no further concerns regarding the toddler's hearing. At three years, the patient remains on maintenance immunomodulator therapy, supervised by her immunologist, with azathioprine 100 mg and sirolimus 1 mg daily. Her recent audiogram showed hearing recovery stabilized to baseline (Figure 3).

## 3. Discussion

### 3.1. Clinical Manifestations and Diagnosis of AIED

AIED is a rare entity first described by McCabe in a case series of 18 patients with progressive bilateral SNHL without identifiable etiology who were steroid responsive [1]. The incidence of AIED is estimated at <5 cases per 100,000 with an estimated prevalence of 45000 in the US [10]. Cochlear neuritis may initially be unilateral and then progress to the other ear, with patients often showing asymmetric audiometric profiles. The pattern of hearing loss can be fluctuant, but generally deteriorates with time. Vestibular involvement is common with 50% of patients experiencing ataxia or positional vertigo [5]. Balance disorders may be underestimated due to the slow development of vestibular dysfunction and compensation by somatosensory and visual systems [11]. Aural fullness and tinnitus are reported in 25–50% of patients [2].

Due to its low incidence combined with our limited understanding of pathophysiology and inability to establish reliable biomarkers, there is no formal diagnostic criterion [12]. AIED is a diagnosis of exclusion in a patient with a suggestive history, favorable response to immunosuppression, or known autoimmune disease. Diagnosis is suspected on audiogram with bilateral sensorineural hearing loss of at least 30 dB at any frequency and evidence of progression in at least one ear on two serial audiograms performed 3 months apart [13, 14]. Serial audiograms are required as the pattern of sensorineural hearing loss may be fluctuant and to monitor response to steroid therapy. The audiometric pattern is an important prognostic indicator for recovery of hearing. Isolated low-frequency hearing loss is associated with better treatment outcomes compared to a flat audiogram or high-frequency losses [15]. The severity of hearing loss prior to treatment has an inverse correlation with recovery. All patients should undergo MRI evaluation to exclude retrocochlear and demyelinating pathology.

### 3.2. Serological Markers in AIED

Laboratory testing can include nonspecific markers of inflammation and autoimmunity including C-reactive protein, erythrocyte sedimentation rate, antinuclear antibody, and complement protein. Specific inner ear antigen tests include the migration inhibition test (MIT), lymphocyte transformation test (LTT), and Western blot analysis. The MIT and LTT have inherent technical difficulties, and results may be heterogeneous [12]. More specific tests for autoreactivity to inner ear antigens such as HSP-70 should be considered. HSP-70 is a constitutively expressed protein that is upregulated in conditions of stress and found in the spiral limbus, spiral prominence, and organ of Corti within the inner ear, and in peripheral organs [6]. Suspicion of AIED should be raised in HSP-70 positive patients; however, it may also be present in the general population and in Meniere's disease—hence, its use as a biomarker is disputed. A diagnostic dilemma between AIED and Meniere's disease exists, and spontaneous recovery of hearing in Meniere's disease may be mistaken as a positive response to immunosuppression in AIED [16]. An extensive immunologic workup is not mandated and should be coordinated by an immunologist with expertise in interpretation of results, as testing can be expensive [17].

### 3.3. Pathogenesis of AIED

Our current understanding regarding the immunological basis of AIED is limited. The inner ear is not immunologically privileged as previously thought, and inflammatory stimuli can recruit immune competent lymphocytes into the labyrinth and endolymphatic sac [18]. Cells enter the scala tympani via the spiral modiolar vein, coordinating labyrinthitis [2, 5, 6, 12]. Studies have shown the presence of autoantibodies directed against inner ear proteins such as HSP-70, cochlin (spiral ganglion), type II collagen (spiral ligament, endolymphatic duct), and KHRI-3 (otolith organs and endolymphatic sac) [2, 6]. Chronic and persistent trafficking of immune cells into the inner ear results in destructive changes including cochlear ischemia and fibrosis, spiral ganglion degeneration, otic capsule spongiosis, and endolymphatic hydrops [2, 5]. Rarely, secondary AIED may induce arthritis of the incudostapedial joint causing conductive hearing loss [6]. Molecular mimicry and shared epitopes between viral antigens and inner ear proteins can cause cross-reactivity, directing an autoimmune response following viral infection [5, 12, 19]. Although plausible, there is currently no evidence of positive viral serology in AIED.

### 3.4. Treatment Modalities in AIED

Corticosteroids remain the primary medical therapy in autoimmune SNHL, with activation of glucocorticoid receptors within the cochlear hair cells causing downregulation of local cytokines and reducing autoantibody production and inflammation [20]. This attenuates cochleitis and hair cell death, with potential recovery of function. Steroids increase microvascular blood flow in the cochlea and reduce endolymphatic hydrops. Almost 70–90% are steroid responsive, although adjunctive immunosuppressive agents may be indicated in relapses of hearing loss during the maintenance or steroid weaning phase [14]. Azathioprine, a purine analog, has demonstrated improvement in hearing in combination with prednisone in a cohort study of 12 patients [21]. We used azathioprine because of compatibility with pregnancy. In later pregnancy after organogenesis, cyclophosphamide or tacrolimus can be considered [22]. Management of vestibular dysfunction and balance disorders involves balance physiotherapy and treatment of the autoimmune process.

Plasmapheresis removes circulating immune complexes and autoantibodies and is an adjunct in managing autoimmune diseases [5]. It can be considered in severe hearing impairment or as salvage treatment in steroid-resistant cases and involves thrice weekly treatment for 2 weeks and then weekly for four weeks [23]. IVIG therapy provides immunoregulatory action by altering complement-mediated destruction, neutralizing autoantibodies, and modulating autoreactive B cells [24].

Nonsteroidal immunomodulators and biologics have been used in treatment in patients who are not candidates for high-dose steroid therapy, who fail first-line steroid therapy, and to encourage steroid weaning. A recent systematic review revealed steroid nonresponders may benefit from biologics, although studies are limited by small cohorts and variable efficacy [20]. TNF-alpha inhibitors such as systemic etanercept 25 mg twice weekly have shown significant improvement in hearing thresholds [25]. Local instillation of infliximab weekly for 4 weeks revealed stable improvement in hearing at 10–38 weeks after treatment [26]. Anakinra, an interleukin-1*β* blocker, demonstrated improvement in hearing thresholds in a clinical trial of 14 patients with AIED who were nonresponders to steroids [27]. Rituximab, a CD20 antagonist, has been shown to reduce corticosteroid dosage and improve symptoms in AIED [28]. It has demonstrated ability to maintain hearing improvement after corticosteroid use [29], but it has limited use after the first trimester of pregnancy [30].

Patients with destructive inner ear changes have poor outcomes, even with maximal medical management [6, 17, 31]. Time sensitive cochlear implantation should be considered in cases when useful hearing is not restored and maintained [31]. Cochlear implantation does not address the underlying disease process and can be challenging in immune-mediated labyrinthitis ossificans and intracochlear fibrosis [32]. Future treatment strategies include gene and stem cell therapy aimed at neuronal preservation within the cochlea and hair cells [33]. In vivo models using human adipose-derived mesenchymal stem cells demonstrated improvement in hearing in AIED [34].

### 3.5. AIED in Pregnancy

Therapeutic decisions in pregnancy are complicated by the potential risk of treatment on maternal and fetal outcomes and the concern for autoimmune targeting of the fetal inner ear. Combination modalities should be incorporated to minimize systemic corticosteroid dosing. Long-term, high-dose steroid therapy has an unfavorable side effect profile. The literature is inconclusive on the safety of prolonged steroid administration in pregnancy, and there is currently insufficient evidence to suggest an increased risk of fetal malformations or gestational diabetes mellitus [35]. Multidisciplinary discussion between otolaryngology, immunology, and MFM facilitates sharing of expertise and ensuring optimal management of the immune-mediated hearing loss without compromising maternal or fetal outcomes. Due to limited evidence on the management of AIED, a collaborative approach is preferred [14].

The obstetrician provides expertise to guide safe dosing of steroids in pregnancy and whether steroid-sparing or steroid-minimizing treatment should be utilized. Alternate options include intratympanic dexamethasone, oral immunomodulator therapy, plasmapheresis, and IVIG [20]. Intratympanic steroid injection can achieve higher perilymph steroid concentrations compared to oral or intravenous administration [36, 37]. Systemic therapy is indicated to ameliorate the underlying multisystem autoimmune response. In pregnancy, there is concern for transplacental transfer of autoantibodies to the fetus-plasmapheresis or IVIG may provide benefit. Similar to our case, one study reported a higher incidence of abnormal auditory brainstem response findings in the children of women who developed AIED during pregnancy [38]. Hence, we must recognize that autoimmune hearing loss in pregnancy may also have implications on the newborn's hearing. There are no data on whether the hearing deficits persisted in childhood.

Hill et al. described the first case of primary AIED in pregnancy, reporting that oral steroids minimized further deterioration in hearing, but the patient did not recover to baseline levels [8]. In that case, the patient presented late and treated only with oral prednisone. In our case, early initiation of intensive steroids, including pulsed methylprednisolone and intratympanic dexamethasone, and multimodal long-term treatment showed great efficacy. Cogan's syndrome, a form of secondary AIED characterized by bilateral cochleovestibulitis and keratitis, has been described in pregnancy in two case reports. One study maintained disease stability with hydroxychloroquine and prednisone, whilst the other treated symptomatic flares with oral and ophthalmic steroids [39, 40].

## 4. Conclusion

AIED is an otologic emergency that rarely manifests during pregnancy and requires urgent treatment, supervised by otolaryngologists, immunologists, and MFM specialists. Multimodal combination therapy is effective and minimizes long-term systemic corticosteroid exposure. We report a rare case of primary AIED in pregnancy with remarkable hearing outcomes following combination (IV, oral, and intratympanic) steroid therapy, augmented by immunomodulators, plasmapheresis, and IVIG.

## Figures and Tables

**Table 1:** CSF studies.

Cerebrospinal fluid analysis		
CSF glucose	2.8 mmol/L	2.2–3.9
CSF protein	0.43 g/L	0.15–0.45
CSF LDH	24 U/L	
CSF cell count		
Leukocytes	36 × 10^6^/L	
Erythrocytes	2 × 10^6^/L	
Polymorphonuclear cells	Nil	
Mononuclear cells	36 × 10^6^/L	
Oligoclonal bands	Positive, CSF restricted	
Culture	No bacterial growth	
Mycobacterial culture	Negative	
Mycobacterial NAT	Negative	
Nucleic acid testing for EBV, VZV CMV, enterovirus, HSV, and polyomavirus	Negative	
Cerebrospinal fluid immunologic tests		
Neuronal antibodies: Purkinje (Yo), PCA 2, ANNA-1, ANNA-2, Ma 1, Ma 2, amphiphysin, CV2, Tr, SOX-1	Negative	
NMDA receptor antibodies	Negative	
CASPR2 antibodies	Negative	
LGI-1 antibodies	Negative	
GABA-B antibodies	Negative	
DPPX antibodies	Negative	
IgLON5 antibodies	Negative	
Albumin	0.21 g/L	0–0.35
Immunoglobulin G	0.03 g/L	0–0.03
IgG/albumin ratio	0.14	0–0.25

**Table 2:** Baseline laboratory tests.

*Full blood count*		
Haemoglobin	127 g/L	115–165
White cell count	5.5 × 10^9^/L	4.0–11.0
Platelets	159 × 10^9^/L	150–400
Haematocrit	0.37 L/L	0.36–0.44
Mean corpuscular volume	87 fL	82–98
Mean corpuscular Hb	30 pg	27–32
Mean corpuscular Hb concentration	345 g/L	300–350
Red cell distribution width	12.3%	11.0–15.0

*Serum biochemistry*		
Sodium	136 mmol/L	135–145
Potassium	3.6 mmol/L	3.2–5.0
Chloride	107 mmol/L	95–110
Bicarbonate	20 mmol/L	22–32
Urea	2.7 mmol/L	2.5–6.5
Creatinine	52 umol/L	45–90
eGFR	>90 mL/min/1.73 m^2^	>90
Calcium	2.42 mmol/L	2.15–2.55
Magnesium	0.83 mmol/L	0.70–1.10
Phosphate	1.09 mmol/L	0.75–1.50
C-reactive protein	<3 mg/L	<3
Erythrocyte sedimentation rate	50 mm/hr	3–19

**Table 3:** Viral and infectious diseases serology.

*Viral serology*		
CMV IgG	Not detected	
CMV IgM	Not detected	
EBV IgG	Detected	
EBV IgM	Equivocal	
HSV1 IgG	Detected	
HSV2 IgG	Not detected	
HSV IgM	Not detected	
Varicella zoster IgG	Detected	
Varicella zoster IgM	Not detected	
HIV Ag/Ab screen	Not detected	
HBsAg	Not detected	
HCV antibody	Not detected	
Ross River virus IgM	Not detected	
Barmah Forest virus IgM	Not detected	
Arbovirus serology	Not detected	

*Infectious disease serology*		
Syphilis EIA total antibody	Nonreactive	
Anti-DNase B titre	<100	<200
Antistreptolysin O titre	91.4 IU/mL	<200
TB gamma interferon	Not detected	

**Table 4:** Metabolic and endocrinologic profile.

Metabolic and endocrine profile		
Iron level	5.9 umol/L	7.0–29.0
Transferrin	2.7 g/L	1.8–3.3
Transferrin saturation	10%	10–45
Ferritin	29 ug/L	15–150
HbA1c%	5.0%	4.0–6.0
Vitamin B12	117 pmol/L	>150
Holotranscobalamin level	61 pmol/L	>36
Serum folate	18.9 nmol/L	>10.0
TSH	2.57 mIU/L	0.40–3.50
Free thyroxine	14.0 pmol/L	9.0–19.0
Angiotensin converting enzyme	32 UL	20–70
Urate	0.18 mmol/L	0.12–0.38
Homocysteine	6.2 umol/L	4.5–13.5
Chromogranin A	1.9 nmol/L	<3.0
Neopterin	12 nmol/L	0–13
Total bile acids	8 umol/L	≤8.0
Apolipoprotein A1	1.68 g/L	1.11–2.09
Apolipoprotein B	1.18 g/L	0.63–1.32
Apolipoprotein A1/B ratio	1.42	0.87–2.85
Lipoprotein EPG	Normal limits	

**Table 5:** Immunologic and cell markers, including flow cytometry profile and genetic studies.

*Cell markers*		
CD19 pan B cell	7%	
CD20 mature B cell	7%	
Kappa light chain	3%	
Lambda light chain	3%	
CD3 T cells	69%	
CD4 helper subset	34%	
CD8 cytotoxic subset	32%	
Flow comment	No evidence of lymphoproliferative disease	

*Immunologic markers*		
Rheumatoid factor	<10 IU/mL	<15
Anti-CCP antibodies	3 U/mL	<5
IgG	8.7 g/L	6.6–15.6
IgA	1.73 g/L	0.75–3.80
IM	1.56 g/L	0.40–3.10
IgE	19 U/mL	<113
IgG1	3.76 g/L	3.92–9.12
IgG2	2.60 g/L	1.50–6.40
IgG3	0.48 g/L	0.25–1.38
IgG4	0.27 g/L	0.04–0.70
C1Q complement component	155 mg/L	118–244
C2 complement	29.9 mg/L	14.0–5.0
C3 complement	1.30 g/L	0.74–1.57
C4 complement	0.28 g/L	0.13–0.41
C5 complement	>200.0 mg/L	100–169
C6 complement	120 mg/L	45–96
C7 complement	>110 mg/L	55–85
C8 complement	172.0 mg/L	112–172
C9 complement	500.0 mg/L	125–265
Haptoglobin	1.53 g/L	0.30–2.15
A1 antitrypsin level	2.38 g/L	0.90–1.90
Serum total protein	63 g/L	46–70
Albumin EPG	36 g/L	37–51
Alpha1 globulin	2.9 g/L	0.9–2.0
Alpha2 globulin	8.9 g/L	2.8–7.7
Beta globulin	8.9 g/L	5.1–14.0
Gamma globulin	6.9 g/L	5.1–14.0
B2 microglobulin	1.9 mg/L	1.0–2.6
Glutamic acid decarboxylase antibody	<5 U/mL	≤5
Islet cell antigen 512 antibodies	<8 U/mL	0–15
Nuclear antibodies	Detected 1 : 640 speckled	
dsDNA antibodies	Pattern	
RNP antibodies	7 IU/mL	0–29
SM (Smith) antigen antibodies	Not detected	
SSA/Ro 60 antibodies	Not detected	
Ro-52/TRIM 21 antibodies	Not detected	
SSB/La antibodies	Not detected	
Scleroderma 70 antibodies	Not detected	
Jo-1 antibodies	Not detected	
Neuronal antibodies: Purkinje	Not detected	
(Yo), PCA 2, ANNA-1, ANNA-2, Ma 1, Ma 2, amphiphysin, CV2, Tr, and SOX-1	Not detected	
Smooth muscle antibodies	Not detected <1 : 40	
Mitochondrial antibodies	Not detected <1 : 40	
Parietal cell antibodies	Not detected <1 : 40	
Intrinsic factor antibody	4 U/mL	<20
Thyroglobulin antibodies	<20 IU/mL	0–60
Thyroid peroxidase antibodies	<10 IU/mL	0–35
Heat shock protein 70 antibodies	Detected A	
Pancreatic islet cell antibodies	Not detected	
Gliadin IgG deamidated antibodies	2 units	<20
Transglutaminase IgA antibodies	<1 U/mL	<4
Neutrophil cytoplasm PR3 and MPO antibodies	Not detected	
Ro-52, Mi-2, Ku, PM-Scl 100, PM-Scl 75, SRP, EJ, OJ, Jo-1, PL-7, and PL-12	Negative	
Zinc transporter 8 antibodies	Negative <10 U/mL	

*Genetic diagnostics*		
Thiopurine methyltransferase genotype	No TMPT variant alleles	
Factor V DNA	No factor V Leiden mutation	

## Data Availability

No data were used to support this study.

## Abbreviations

AIED: Autoimmune inner ear disease
CSF: Cerebrospinal fluid
ENT: Ear, nose, and throat
HSP: Heat shock protein
HSV: Herpes simplex virus
IVIG: Intravenous immunoglobulin
MRI: Magnetic resonance imaging
MFM: Maternal-fetal medicine
MIT: Migration inhibition test
LIT: Lymphocyte transformation test
SNHL: Sensorineural hearing loss
TNF: Tumour necrosis factor.

## Supplementary Materials

Supplementary Materials The CARE checklist for presentation of a case report is provided.
