# Supplementary figures and images for: Combination Immunosuppressive Therapy in Primary Autoimmune Inner Ear Disease in Pregnancy
Source: Case Rep Otolaryngol. 2022 Mar 18;2022:9210780. doi: 10.1155/2022/9210780 (PMC8956394; doi:10.1155/2022/9210780)

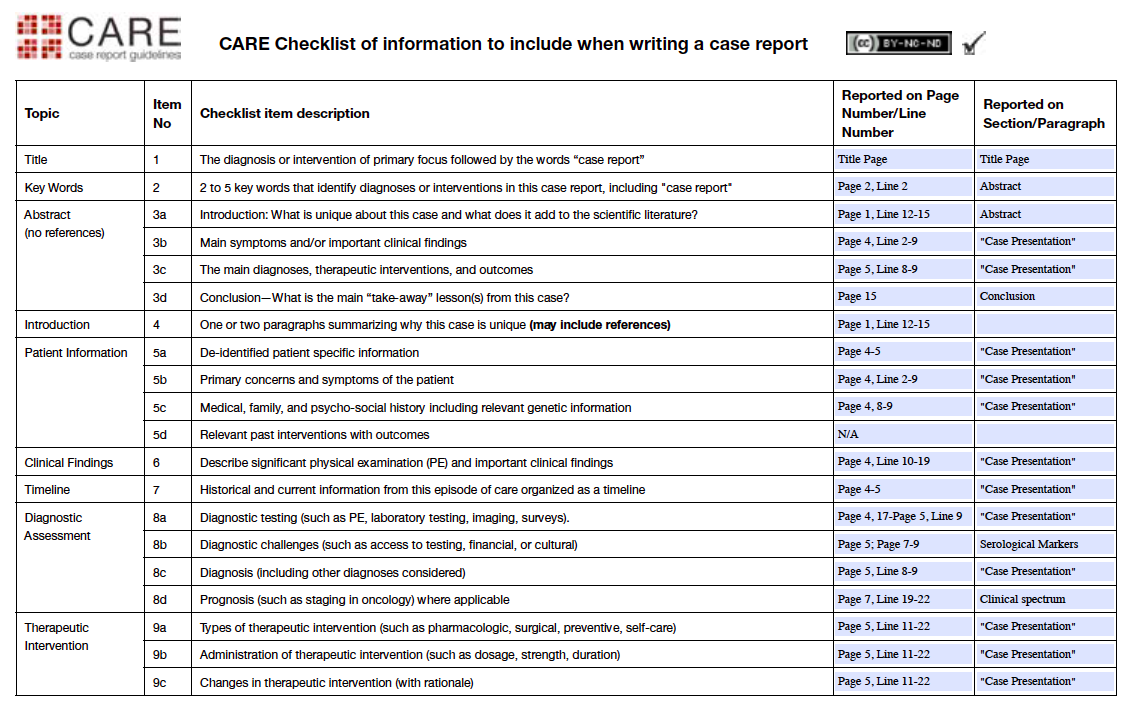


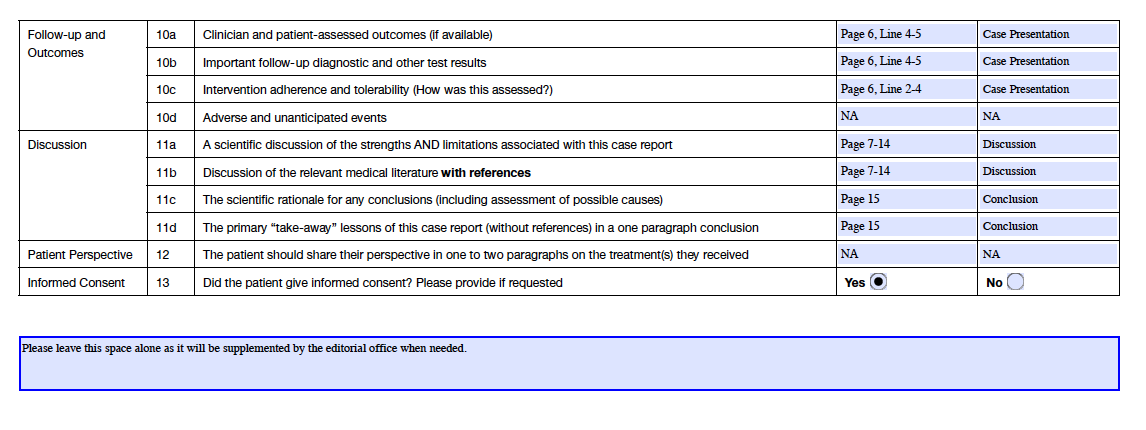

Supplement: Supplementary Materials — The CARE checklist for presentation of a case report is provided. [file 9210780.f1.docx]
